# Prevalence of classic, MLB-clade and VA-clade Astroviruses in Kenya and The Gambia

**DOI:** 10.1186/s12985-015-0299-z

**Published:** 2015-05-15

**Authors:** Caroline T. Meyer, Irma K. Bauer, Martin Antonio, Mitchell Adeyemi, Debasish Saha, Joseph O. Oundo, John B. Ochieng, Richard Omore, O. Colin Stine, David Wang, Lori R. Holtz

**Affiliations:** Washington University School of Medicine, Saint Louis, MO USA; Medical Research Council Unit, Banjul, The Gambia; Center for International Health, Department of Preventive and Social Medicine, Dunedin School of Medicine, University of Otago, Dunedin, New Zealand; Kenya Medical Research Institute/Centers for Disease Control and Prevention, Kisumu, Kenya; University of Maryland School of Medicine, Baltimore, MD USA

**Keywords:** Astrovirus, MLB, VA, Diarrhea, Gastroenteritis

## Abstract

**Background:**

Infectious diarrhea leads to significant mortality in children, with 40 % of these deaths occurring in Africa. Classic human astroviruses are a well-established etiology of diarrhea. In recent years, seven novel astroviruses have been discovered (MLB1, MLB2, MLB3, VA1/HMO-C, VA2/HMO-B, VA3/HMO-A, VA4); however, there have been few studies on their prevalence or potential association with diarrhea.

**Methods:**

To investigate the prevalence and diversity of these classic and recently described astroviruses in a pediatric population, a case–control study was performed. Nine hundred and forty nine stools were previously collected from cases of moderate-to-severe diarrhea and matched controls of patients less than 5 years of age in Kenya and The Gambia. RT-PCR screening was performed using pan-astrovirus primers.

**Results:**

Astroviruses were present in 9.9 % of all stool samples. MLB3 was the most common astrovirus with a prevalence of 2.6 %. Two subtypes of MLB3 were detected that varied based on location in Africa. In this case–control study, Astrovirus MLB1 was associated with diarrhea in Kenya, whereas Astrovirus MLB3 was associated with the control state in The Gambia. Classic human astrovirus was not associated with diarrhea in this study. Unexpectedly, astroviruses with high similarity to Canine Astrovirus and Avian Nephritis Virus 1 and 2 were also found in one case of diarrhea and two control stools respectively.

**Conclusions:**

Astroviruses including novel MLB- and VA-clade members are commonly found in pediatric stools in Kenya and The Gambia. The most recently discovered astrovirus, MLB3, was the most prevalent and was found more commonly in control stools in The Gambia, while astrovirus MLB1 was associated with diarrhea in Kenya. Furthermore, a distinct subtype of MLB3 was noted, as well as 3 unanticipated avian or canine astroviruses in the human stool samples. As a result of a broadly reactive PCR screen for astroviruses, new insight was gained regarding the epidemiology of astroviruses in Africa, where a large proportion of diarrheal morbidity and mortality occur.

## Background

Diarrheal disease is a leading cause of death in children under five years of age worldwide [1], and a high proportion of these deaths, approximately 40 %, occur in Africa [2]; however this population has been rarely studied [3]. Additionally, infectious diarrhea causes significant morbidity including growth faltering, malnutrition and impaired cognitive development [4].

Classic human astroviruses (HAstV) are a well-established etiology of viral gastroenteritis, causing approximately 2 % to 8 % of childhood diarrhea in Africa [5–10]. All 8 serotypes of HAstV have been associated with diarrhea. Since 2008, seven highly divergent astroviruses (MLB1, MLB2, MLB3, VA1/HMO-C, VA2/HMO-B, VA3/HMO-A, VA4) have been discovered in human stool samples [11–16]. To date, there have been few prevalence studies and no definitive disease association established for these viruses.

MLB1 was the first of these to be discovered and has been detected in stools across the world including Australia, Asia, Africa, North America and Europe [11, 13, 15–22]. Furthermore a seroepidemiologic study of MLB1 demonstrated that primary exposure occurs in childhood and that seropositivity reached 100 % by adulthood [23]. One case–control study of MLB1 has been reported of Indian children that did not show an association of MLB1 with diarrhea [24]. MLB2 was initially discovered in stool, and MLB2 viremia in a febrile child has been described [25]. MLB3 was described recently in India in 2013 and found in 0.6 % of stools sampled [12].

VA1/HMO-C was first identified in an unexplained outbreak of gastroenteritis [14] and has since been detected in the brain of multiple immunocompromised patients with encephalitis [26–28]. This virus has been found to have a seroprevalence of 65 % in adults [29]. VA2/HMO-A and VA3/HMO-B have been described in stools from Nigeria, Pakistan, and India, while VA2/HMO-A has additionally been found in stools from the United States, China and Egypt [11, 13, 18, 21]. VA4 was recently discovered in 2 stool samples from Nepal [12].

Due to the limited data on these novel astroviruses, the objective of this study was to explore the prevalence and diversity of astroviruses in a pediatric population using cases of moderate-severe diarrhea (MSD) and matched controls in Kenya and The Gambia.

## Results

Astroviruses were present in 9.9 % of all samples and represented primarily a mixture of classic, MLB and VA astroviruses (MLB3 2.6 %, HAstV 2.5 %, MLB1 1.7 %) (Table 1). MLB3 was the most prevalent astrovirus, followed by HAstV and MLB1.

**Table 1:** Astrovirus Screening Results

	Kenya			The Gambia			All samples			
	Cases	Controls	p	Cases	Controls	p	Cases	Controls	p	Total
	n = 181	n = 181		n = 266	n = 321		n = 447	n = 502		n = 949
HAstV	7	4	0.3270	5	8	0.4609	12	12	0.9432	24
MLB1	**11**	**1**	0.0217*	2	2	1.000	**13**	**3**	0.0221*	16
MLB2	0	1	0.9907	2	7	0.2722	2	8	0.1808	10
MLB3	1	6	0.9864	**3**	**15**	0.0421*	**4**	**21**	0.0071*	25
VA1	0	0	N/A	0	1	0.9857	0	1	0.9857	1
VA2	2	2	1.0000	4	6	0.9435	6	8	0.9569	14
VA3	0	0	N/A	0	0	N/A	0	0	N/A	0
VA4	0	0	N/A	0	0	N/A	0	0	N/A	0
Total^†^	21	14	N/A	16	39	N/A	37	53	N/A	90

HAstV- Human Astrovirus

N/A- Not applicable

*Significant at p < 0.05

^†^Total does not include additional astroviruses including Canine Astrovirus, Avian Nephritis Virus 1 and 2, and VA5

**Fig. 1:**
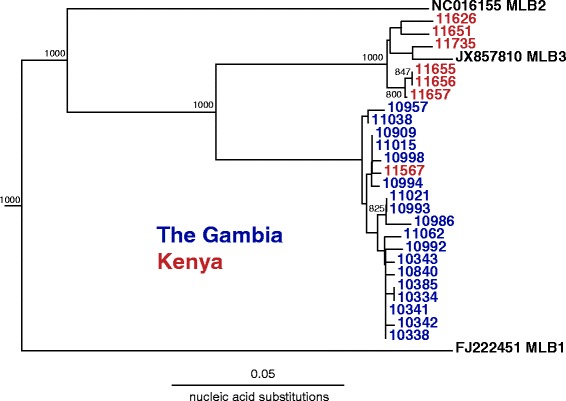
Phylogenetic Analysis of MLB3 amplicons. Amplicons measuring 409 nucleotides in length from ORF 1b were used for phylogenetic analysis. Significant bootstrap values (>700) are shown. Scale bar represents the number of nucleic acid differences. GenBank accession numbers or sample numbers are listed for reference

MLB3 was detected more frequently in controls than in diarrhea cases in The Gambia (4.7 % controls vs 1.1 % cases; p = 0.0421) as well as overall (4.2 % controls vs 0.9 % cases; p = 0.0071). Sequencing of the amplicons from the MLB3 positive samples from 6 of 7 Kenyan samples yielded sequences that shared 97-99 % identity with the reference MLB3 strain. However, the seventh Kenyan sample and all 18 positive samples from The Gambia shared only 88–89 % identity to the reference MLB3 strain, demonstrating that a previously unrecognized diversity of MLB3 subtypes exists (Fig. 1).

MLB1 was significantly associated with diarrhea in Kenya (6.1 % cases vs 0.6 % controls; p = 0.0217). All of the MLB1 positive stools in Kenya occurred in a 4 month timeframe of stool collection between May-August of 2008 with 7 of the 12 positive samples collected within 12 days. By contrast, prevalence of MLB1 was lower in The Gambia, 3 of the 4 detections occurred in March and April, and no association with diarrhea was observed (0.8 % cases vs. 0.6 % controls). There was no difference in the prevalence of classic astroviruses between cases and controls (2.7 % cases vs. 2.4 % controls). VA-clade viruses were detected at low prevalence (VA1 and VA2) or not at all (VA3 and VA4).

The results of previous microbiologic testing for 17 diarrhea pathogens for these samples [3] were used to determine if any of the known pathogens were detected with any astrovirus more frequently than by chance. Prevalence of *Campylobacter jejuni* infections in the aggregate Kenya and The Gambia population was 4.5 % (5.8 % of cases and 3.4 % of controls) (Table 2). Co-detection of MLB1 and *Campylobacter jejuni* was significant; 5 of the 16 stools positive for MLB1 were also positive for *C. jejuni* (p = 0.0005). All five of the samples with both MLB1 and *C. jejuni* were diarrhea cases.

**Table 2:** *Campylobacter jejuni* and MLB1 Co-detection

	MLB1 Negative	MLB1 Positive	Total
*Campylobacter* Negative	895 (94.3 %)	11 (1.2 %)	906
*Campylobacter* Positive	38 (4.0 %)	5 (0.5 %)*	43
Total	933	16	949

*Co-detection of MLB1 and *Campylobacter jejuni* was significant at p = 0.0005 using Fisher exact test, 95 % CI (2.757–35.260)

**Fig. 2:**
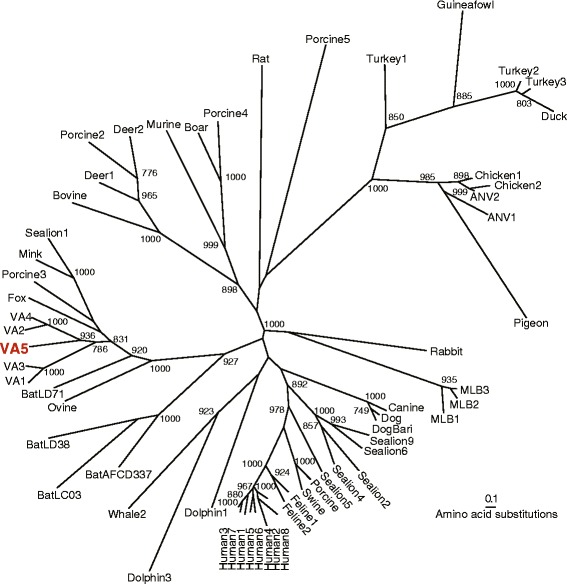
Phylogenetic Analysis of Astrovirus ORF2 capsid protein. The complete amino acid sequence from ORF2 was used for phylogenetic analysis. Significant bootstrap values (>700) are shown. Scale bar represents the number of amino acid differences. ANV: Avian Nephritis Virus

Sequencing of the pan-astrovirus primer derived amplicons also yielded several unexpected results. A 415 nucleotide (nt) amplicon [GenBank: KJ777800] from a Kenyan case stool shared 95 % nucleotide identity to Canine astrovirus strain 915 [GenBank: JX878480]. In addition, a 439 nt amplicon [GenBank: KJ777802] from a Kenyan control stool shared 92 % identity to Avian Nephritis Virus 2 [GenBank: HQ188698] [30], while a 439 nt amplicon [GenBank: KJ777801] from a Gambian control sample shared 94 % identity to Avian Nephritis Virus 1 [GenBank: AB033998] [31]. Finally, a 433 nt amplicon from a control sample from the Gambia shared only 77 % nucleotide identity to VA4. We sequenced the nearly-complete genome of 6519 nt [GenBank: KJ656124], which we tentatively named astrovirus VA5 (Fig. 2). Further analysis demonstrated that this sequence shared 94 % nucleotide identity to a very recently published sequence Human astrovirus BF34 [GenBank: KF859964] [32]. VA5 contains characteristic astrovirus motifs such as a heptameric slippery sequence (AAAAAAC) and a stem-loop II in the 3′ UTR.

## Discussion

In summary, astroviruses are commonly found in pediatric stool samples in Kenya and The Gambia. While there have been a few reports describing the prevalence of recently identified astroviruses in African countries such as Egypt and Nigeria [13, 18] there have been no studies from Kenya or The Gambia. Astrovirus MLB3 was recently discovered in India in 2013 [12] and has not been previously detected in Africa; however, it was the most frequently detected of all of the astroviruses in this study.

This is only the second study reporting MLB3 and the first case–control study of MLB3 performed. Compared to the initial study in India, MLB3 was detected at a prevalence four times higher in Africa [12]. Furthermore, there is a novel diversity of MLB3 viruses, as a distinct subtype of MLB3 that differed by > 10 % from the reference MLB3 was detected. Prior studies of the 8 classic human astrovirus serotypes have also shown geographic strain variation [33].

Interestingly, MLB3 was negatively associated with moderate-severe diarrhea in The Gambia. Although viruses are generally associated with undesirable consequences, a recent animal model showed that enteric viruses may also have beneficial functions [34]. In the animal model, murine norovirus provided protection against mucosal damage and supported intestinal homeostasis in germ-free and antibiotic-treated mice. The association of MLB3 with the control state, may indicate that MLB3 can similarly provide protection from diarrhea in this population. Alternatively, MLB3 may be a pathogen of another organ system and affected by diarrhea-associated changes in the gut.

There was a positive association between MLB1 and diarrhea in this matched case–control study in Kenya, raising the possibility that MLB1 may play a role in the etiology of diarrhea. However, no association was observed in The Gambia. In Kenya, there was a clustering of positive stools over a 12 day time period. This could indicate an outbreak of MLB1 leading to diarrhea and increased prevalence that was not captured in The Gambia. The association of MLB1 with diarrhea also differed from a prior case–control study in India [24]. Although the aggregate analysis of Kenya and The Gambia together still yields a statistically significant association with diarrhea overall (p = 0.0221), the different results from the two sites renders it difficult to make definitive conclusions.

Clearly the two geographic sites yielded distinct prevalence rates for both MLB1 and MLB3. Geographic variation of eukaryotic viruses occurs [35] and likely contributed to our findings. These observations underscore the need for additional studies in diverse geographic sites to better define the prevalence of these novel astroviruses.

This study showed a significant association between MLB1 and *C. jejuni. Campylobacter* is typically a food-borne or zoonotic disease [36], while classic human astrovirus is most commonly transmitted by human to human contact [33]. Although the route of transmission of MLB1 is currently unknown, the high rate of co-detection could be due to similar transmission of MLB1 and *Campylobacter* through food or water. Previous studies have shown that mixed infections with *Campylobacter* and other pathogens are common [37, 38], and specific virus-induced cell alterations enhanced the invasiveness of *C. jejuni* in a cell culture model [39]. These observations raise the question of whether there may be synergistic effects between these agents and whether those effects play a role in the etiology of diarrhea, as all co-detections occurred in cases with diarrhea.

Although HAstV is a well-established cause of diarrhea, it was not associated with moderate-severe diarrhea in this study. This observation may be due to the exclusion of mild cases of diarrhea typically associated with HAstV infection. This result also reflects some of the challenges of using the case–control approach for disease attribution as other well-defined human diarrhea pathogens have also failed in single case–control studies [40, 41].

Unexpectedly, there were sequences in human stool with high identity to Avian Nephritis Virus 1 and 2 and Canine Astrovirus. These observations may be the result of ingestion of infected meat, as this phenomenon has been described for plant viruses [17, 42] or may be related to environmental exposure to animals. Zoonotic infection has been postulated to occur in astroviruses due to the frequent occurrence of recombination events and wide range of species infected [43] and is another possibility. In addition, a novel astrovirus VA5, similar to the recently reported HAstV-BF34 was identified [32]. It was only the use of broadly reactive PCR primers in this study that enabled the detection of these unexpected or novel agents. Prior studies of the prevalence of classic HAstV used primers that targeted specifically Human Astroviruses 1–8. Those primers could not detect viruses such as Avian Nephritis Virus or Canine Astrovirus. With the dramatic increase the number of astrovirus species discovered, further optimization of consensus astrovirus primers and their application to analyze human cohorts may uncover much more about the types and frequency of astroviruses found in human stool.

## Conclusions

In conclusion, astroviruses including the novel MLB- and VA-clades are commonly found in pediatric stools in Kenya and The Gambia and a wide range of astroviruses was detected in this population. One of the most recently discovered astroviruses, MLB3 was also the most prevalent. MLB1 was associated with diarrhea in Kenya, while MLB3 was found more commonly in the control stools in The Gambia. There is diversity within the species of MLB3. Unexpected animal astrovirus species were detected in human stool through the use of broadly reactive PCR primers. Additional studies are needed to better understand the role of these novel astroviruses in human health and disease.

## Methods

### Samples

This investigation utilized 949 stool samples from cases of moderate-severe diarrhea and matched controls aged 0–59 months participating in the Global Enteric Multicenter Study (GEMS) at two rural sites in Kenya and The Gambia [3]. Samples were collected from Kenya between April 2008 and December 2008 and from The Gambia between March 2008 and April 2009. The study was approved by ethics committees at the University of Maryland, Baltimore, MD, USA, and at both field sites.

Cases of moderate-severe diarrhea were defined as acute (onset within 7 days) with ≥ 3 loose stools within 24 hours and 1 of the following criteria: sunken eyes, loss of skin turgor, intravenous hydration prescribed, dysentery, or admission to hospital. For every enrolled case, one to three matched control children was enrolled. Controls were matched to every case by age (±2 months for patients aged 0–23 months and ±4 months for patients aged 24–59 months), sex and residence (same or nearby village as the patient with diarrhea) (Table 3). Controls were enrolled within 14 days of the index case and were excluded if they had diarrhea in the prior 7 days. All stools were previously screened for an array of bacterial, parasitic, and viral pathogens [44].

**Table 3:** Patient Demographics

	Kenya			The Gambia			All samples			
	Cases	Controls	p	Cases	Controls	p	Cases	Controls	p	Total
	n = 181	n = 181		n = 266	n = 321		n = 447	n = 502		n = 949
Age (mos)	17.4	17.5	0.985	16.4	18.0	0.0626	16.8	17.8	0.212	17.1
Gender (M)	55.8 %	55.8 %	1.000	56.4 %	55.5 %	0.820	56.2 %	55.6 %	0.859	55.8 %

In total, 362 stools from Kenya (181 cases, 181 controls) and 587 stools from The Gambia (266 cases, 321 controls) were available for analysis. A Welch two sample *t*-test was used to examine for differences in age between cases and controls. To assess for differences in gender between cases and controls a chi square test was performed. To determine if presence of specific astroviruses was associated with diarrhea a conditional logistic regression was performed. To analyze if there were significant co-infections with astroviruses and other known pathogens Fisher exact test was used. P values <0.05 were determined to be significant. All statistical analyses were done in SAS 9.3 (Carey, NC).

### Screening

RT-PCR screening of nucleic acid extracted from stool filtrates was performed using a one-step kit (Qiagen) with pan-astrovirus primers SF0073 (5′-GATTGGACTCGATTTGATGG-3′) and SF0076 (5′-CTGGCTTAACCCACATTCC-3′) that target the RNA-dependent RNA polymerase as previously described [11]. Positive samples were then screened using primers designed to target the capsid region of classic human astroviruses [Mon269 (5′-CAACTCAGGAAACAGGGTGT-3′) and Mon270 (5′-TCAGATGCATTGTCATTGGT-3′)] and MLB1 [SF0053 (5′-CTGTAGCTCGTGTTAGTCTTAACA-3′) and SF0061(5′-GTTCATTGGCACCATCAGAAC-3′)] [11]. Pan-astrovirus derived amplicons from samples negative in both secondary screens were cloned into pCR4-TOPO vector (Invitrogen) and Sanger sequenced. Sequences were compared to the GenBank nr/nt database on May 7, 2014 and deposited [Genbank: KJ807476 to KJ807529, KJ777800 to KJ777802].

### Full genome sequencing

Deep-sequencing was performed on the stool sample containing the newly described virus VA5 using the Roche Titanium FLX platform [14]. 1004 viral reads were identified using Virushunter [45] and assembled using Newbler generating a contig of 6519 nucleotides. The contig was confirmed by RT-PCR and 3′ rapid amplification of cDNA ends. The complete 5′ UTR was not successfully sequenced after multiple attempts. This 6519 base pair sequence was deposited in Genbank [Genbank: KJ656124].

### Phylogenetic analysis

MLB3 nucleic acid amplicon sequences were aligned and maximum-likelihood trees were created by CLUSTALX version 2.1 with 1000 bootstrap replicates. Phylogenetic analysis of the recently described VA5 was performed by aligning amino acid sequences from the complete ORF2 with MUSCLE. Best-fit model was determined using Prottest (version 2.4) [46], and maximum-likelihood trees were generated using Phyml (version 3.0) [47].

Accession numbers for astrovirus sequences used in Fig. 2 are as follows: Turkey Astrovirus 1: CAB95007; Turkey Astrovirus 2: NP987088; Turkey Astrovirus 3: AAV37187; Duck Astrovirus: ACN82429; Guinea Fowl Astrovirus: JN985815; Chicken Astrovirus 1: NP620618; Chicken Astrovirus 2: BAB21617; Avian Nephritis Virus 1: HM029238; Avian Nephritis Virus 2: AEB15604; Pigeon Astrovirus: CBY02488; Rabbit Astrovirus: AEV92822; Astrovirus MLB1: YP002290968; Astrovirus MLB2: YP004934010; Astrovirus MLB3: JX857870; Canine Astrovirus: AEX00102; Dog Astrovirus: ADL27736; DogBari Astrovirus: HM045005; Sealion Astrovirus 1: ACR54272; Sealion Astrovirus 2: ACR54274; Sealion Astrovirus 4: JN420351; Sealion Astrovirus 5: JN420352; Sealion Astrovirus 6: JN420353; Sealion Astrovirus 9: JN420356; Porcine Astrovirus: BAA90309; Porcine Astrovirus 2: AER30006; Porcine Astrovirus 3: NC019494; Porcine Astrovirus 4: JF713713; Porcine Astrovirus 5: JF713711; Swine Astrovirus: ADV16836; Feline Astrovirus 1: AAC13556; Feline Astrovirus 2: NC022249; Human Astrovirus 1: NP059444; Human Astrovirus 2: L13745; Human Astrovirus 3: AAD17224; Human Astrovirus 4: AAY84779; Human Astrovirus 5: AAY46274; Human Astrovirus 6: CAA86616; Human Astrovirus 7: AAK31913; Human Astrovirus 8: AAF85964; Dolphin Astrovirus 1: ACR54280; Dolphin Astrovirus 3: HQ668129; Whale Astrovirus 2: HQ668143; Bat Astrovirus AFCD337: ACF75865; Bat Astrovirus LC03: ACN88720; Bat Astrovirus LD38: ACN88708; Bat Astrovirus LD71: ACN88712; Ovine Astrovirus: CAB95004; Astrovirus VA1: NC013060; Astrovirus VA2: GQ502193; Astrovirus VA3: JX857868; Astrovirus VA4: JX857869; Astrovirus VA5: KJ656124; Fox Astrovirus: KC692365; Mink Astrovirus: NP795336; Bovine Astrovirus B76: AED89609; Deer Astrovirus 1: HM447045; Deer Astrovirus 2: HM447046; Wild boar Astrovirus: AEZ67026; Murine Astrovirus: NC018702; Rat Astrovirus: HM450382.

## Abbreviation

HAstV: Human Astrovirus

## References

[1] Liu L, Johnson HL, Cousens S, Perin J, Scott S, Lawn JE (2012). Global, regional, and national causes of child mortality: an updated systematic analysis for 2010 with time trends since 2000. Lancet.

[2] Boschi-Pinto C, Velebit L, Shibuya K (2008). Estimating child mortality due to diarrhoea in developing countries. Bull World Health Organ.

[3] Kotloff KL, Nataro JP, Blackwelder WC, Nasrin D, Farag TH, Panchalingam S (2013). Burden and aetiology of diarrhoeal disease in infants and young children in developing countries (the Global Enteric Multicenter Study, GEMS): a prospective, case–control study. Lancet.

[4] Petri WA, Miller M, Binder HJ, Levine MM, Dillingham R, Guerrant RL (2008). Enteric infections, diarrhea, and their impact on function and development. J Clin Invest.

[5] Platts-Mills JA, Gratz J, Mduma E, Svensen E, Amour C, Liu J (2014). Association between stool enteropathogen quantity and disease in Tanzanian children using TaqMan array cards: a nested case–control study. Am J Trop Med Hyg.

[6] Kiulia NM, Mwenda JM, Nyachieo A, Nyaundi JK, Steele AD, Taylor MB (2007). Astrovirus infection in young Kenyan children with diarrhoea. J Trop Pediatr.

[7] Cunliffe NA, Dove W, Gondwe JS, Thindwa BD, Greensill J, Holmes JL (2002). Detection and characterisation of human astroviruses in children with acute gastroenteritis in Blantyre, Malawi. J Med Virol.

[8] Pennap G, Pager CT, Peenze I, de Beer MC, Kwaga JK, Ogalla WN (2002). Epidemiology of astrovirus infection in Zaria, Nigeria. J Trop Pediatr.

[9] Basu G, Rossouw J, Sebunya TK, Gashe BA, de Beer M, Dewar JB (2003). Prevalence of rotavirus, adenovirus and astrovirus infection in young children with gastroenteritis in Gaborone, Botswana. East Afr Med J.

[10] Marx FE, Taylor MB, Grabow WOK (1998). The prevalence of human astrovirus and enteric adenovirus infection in South African patients with gastroenteritis. South Afr J Epidemiol Infect.

[11] Finkbeiner SR, Holtz LR, Jiang Y, Rajendran P, Franz CJ, Zhao G (2009). Human stool contains a previously unrecognized diversity of novel astroviruses. Virol J.

[12] Jiang H, Holtz LR, Bauer I, Franz CJ, Zhao G, Bodhidatta L (2013). Comparison of novel MLB-clade, VA-clade and classic human astroviruses highlights constrained evolution of the classic human astrovirus nonstructural genes. Virology.

[13] Kapoor A, Li L, Victoria J, Oderinde B, Mason C, Pandey P (2009). Multiple novel astrovirus species in human stool. J Gen Virol.

[14] Finkbeiner SR, Li Y, Ruone S, Conrardy C, Gregoricus N, Toney D (2009). Identification of a novel astrovirus (astrovirus VA1) associated with an outbreak of acute gastroenteritis. J Virol.

[15] Finkbeiner SR, Le BM, Holtz LR, Storch GA, Wang D (2009). Detection of newly described astrovirus MLB1 in stool samples from children. Emerg Infect Dis.

[16] Finkbeiner SR, Kirkwood CD, Wang D (2008). Complete genome sequence of a highly divergent astrovirus isolated from a child with acute diarrhea. Virol J.

[17] Finkbeiner SR, Allred AF, Tarr PI, Klein EJ, Kirkwood CD, Wang D (2008). Metagenomic analysis of human diarrhea: viral detection and discovery. PLoS Pathog.

[18] Ahmed SF, Sebeny PJ, Klena JD, Pimentel G, Mansour A, Naguib AM (2011). Novel astroviruses in children, Egypt. Emerg Infect Dis.

[19] Banyai K, Meleg E, Moschidou P, Martella V (2010). Detection of newly described astrovirus MLB1 in stool samples from children. Emerg Infect Dis.

[20] Medici MC, Tummolo F, Calderaro A, Elia G, Banyai K, De Conto F (2014). MLB1 astrovirus in children with gastroenteritis, Italy. Emerg Infect Dis.

[21] Wang Y, Li Y, Jin Y, Li DD, Li X, Duan ZJ (2013). Recently identified novel human astroviruses in children with diarrhea, China. Emerg Infect Dis.

[22] Matsumoto T, Wangchuk S, Tshering K, Yahiro T, Zangmo S, Dorji T, et al. Complete Genome Sequences of Two Astrovirus MLB1 Strains from Bhutanese Children with Diarrhea. Genome Announc. 2013;1. 10.1128/genomeA.00485-13 PMC3735058 23887913

[23] Holtz LR, Bauer IK, Jiang H, Belshe R, Freiden P, Schultz-Cherry SL (2014). Seroepidemiology of astrovirus MLB1. Clin Vaccine Immunol.

[24] Holtz LR, Bauer IK, Rajendran P, Kang G, Wang D (2011). Astrovirus MLB1 is not associated with diarrhea in a cohort of Indian children. PLoS One.

[25] Holtz LR, Wylie KM, Sodergren E, Jiang Y, Franz CJ, Weinstock GM (2011). Astrovirus MLB2 viremia in febrile child. Emerg Infect Dis.

[26] Quan PL, Wagner TA, Briese T, Torgerson TR, Hornig M, Tashmukhamedova A (2010). Astrovirus encephalitis in boy with X-linked agammaglobulinemia. Emerg Infect Dis.

[27] Naccache SN, Peggs KS, Mattes FM, Phadke R, Garson JA, Grant P (2015). Diagnosis of neuroinvasive astrovirus infection in an immunocompromised adult with encephalitis by unbiased next-generation sequencing. Clin Infect Dis.

[28] Brown JR, Morfopoulou S, Hubb J, Emmett WA, Ip W, Shah D (2015). Astrovirus VA1/HMO-C: An Increasingly Recognized Neurotropic Pathogen in Immunocompromised Patients. Clin Infect Dis.

[29] Burbelo PD, Ching KH, Esper F, Iadarola MJ, Delwart E, Lipkin WI (2011). Serological studies confirm the novel astrovirus HMOAstV-C as a highly prevalent human infectious agent. PLoS One.

[30] Pantin-Jackwood MJ, Strother KO, Mundt E, Zsak L, Day JM, Spackman E (2011). Molecular characterization of avian astroviruses. Arch Virol.

[31] Imada T, Yamaguchi S, Mase M, Tsukamoto K, Kubo M, Morooka A (2000). Avian nephritis virus (ANV) as a new member of the family Astroviridae and construction of infectious ANV cDNA. J Virol.

[32] Phan TG, Nordgren J, Ouermi D, Simpore J, Nitiema LW, Deng X (2014). New astrovirus in human feces from Burkina Faso. J Clin Virol.

[33] Bosch A, Pinto RM, Guix S (2014). Human astroviruses. Clin Microbiol Rev.

[34] Kernbauer E, Ding Y, Cadwell K (2014). An enteric virus can replace the beneficial function of commensal bacteria. Nature.

[35] Holtz LR, Cao S, Zhao G, Bauer IK, Denno DM, Klein EJ (2014). Geographic variation in the eukaryotic virome of human diarrhea. Virology.

[36] Allos BM (2001). Campylobacter jejuni Infections: update on emerging issues and trends. Clin Infect Dis.

[37] Mason J, Iturriza-Gomara M, O'Brien SJ, Ngwira BM, Dove W, Maiden MC (2013). Campylobacter infection in children in Malawi is common and is frequently associated with enteric virus co-infections. PLoS One.

[38] Glass RI, Stoll BJ, Huq MI, Struelens MJ, Blaser M, Kibriya AK (1983). Epidemiologic and clinical features of endemic Campylobacter jejuni infection in Bangladesh. J Infect Dis.

[39] Konkel ME, Joens LA (1990). Effect of enteroviruses on adherence to and invasion of HEp-2 cells by Campylobacter isolates. Infect Immun.

[40] Bodhidatta L, McDaniel P, Sornsakrin S, Srijan A, Serichantalergs O, Mason CJ (2010). Case–control study of diarrheal disease etiology in a remote rural area in Western Thailand. Am J Trop Med Hyg.

[41] Hien BT, Trang do T, Scheutz F, Cam PD, Molbak K, Dalsgaard A (2007). Diarrhoeagenic Escherichia coli and other causes of childhood diarrhoea: a case–control study in children living in a wastewater-use area in Hanoi, Vietnam. J Med Microbiol.

[42] Zhang T, Breitbart M, Lee WH, Run JQ, Wei CL, Soh SW (2006). RNA viral community in human feces: prevalence of plant pathogenic viruses. PLoS Biol.

[43] De Benedictis P, Schultz-Cherry S, Burnham A, Cattoli G (2011). Astrovirus infections in humans and animals - molecular biology, genetic diversity, and interspecies transmissions. Infect Genet Evol.

[44] Panchalingam S, Antonio M, Hossain A, Mandomando I, Ochieng B, Oundo J (2012). Diagnostic microbiologic methods in the GEMS-1 case/control study. Clin Infect Dis.

[45] Zhao G, Krishnamurthy S, Cai Z, Popov VL, Da Rosa AP T, Guzman H (2013). Identification of novel viruses using VirusHunter–an automated data analysis pipeline. PLoS One.

[46] Abascal F, Zardoya R, Posada D (2005). ProtTest: selection of best-fit models of protein evolution. Bioinformatics.

[47] Guindon S, Dufayard JF, Lefort V, Anisimova M, Hordijk W, Gascuel O (2010). New algorithms and methods to estimate maximum-likelihood phylogenies: assessing the performance of PhyML 3.0. Syst Biol.

